# Editorial: Towards a psychophysiological approach in physical activity, exercise, and sports

**DOI:** 10.3389/fpsyg.2023.1191670

**Published:** 2023-04-27

**Authors:** Pedro Forte, José E. Teixeira, Daniel Leite Portella, Diogo Monteiro

**Affiliations:** ^1^CI-ISCE, Higher Institute of Educational Sciences of the Douro, Penafiel, Portugal; ^2^Research Center in Sports, Health and Human Development, Covilhã, Portugal; ^3^Department of Sports Sciences, Instituto Politécnico de Bragança, Bragança, Portugal; ^4^Department of Sports Sciences, Polithecnic Institute of Guarda, Guarda, Portugal; ^5^Universidade Municipal de São Caetano do Sul, São Caetano do Sul, Brazil; ^6^ESECS - Polytechnique of Leiria, Leiria, Portugal

**Keywords:** psychophysiological, physical activity, exercise, sport, performance

In recent years, a psychophysiological perspective on physical activity, exercise, and sports research has become more popular. Given the strong connections between psychological and physiological aspects, as well as mental fatigue and diseases, psychophysiological and psychological explanations are required. Moreover, human movement and sports performance can also be affected by psychological factors, whereby the regulation of physical capabilities is significantly influenced by the psychophysiological system. It is possible to explain how physical activity and exercise insights are controlled and managed by pacing behavior, decision-making, self-regulation, and effort perception. This Research Topic's goal was to discuss the theoretical and methodological assumptions of the psychophysiological perspective on physical activity, exercise, and sports. In this research area, technical, tactical, physiological, cognitive, and psychological topics were explored in relation to other aspects that affect sports and exercise performance, while the effects of psychological factors on training and performance needs were evaluated. Training interventions assessing acute and chronic adaptations using evaluation and testing procedures make them one of the most important topics in this Research Topic. Research on sports coaching and its relations with mental health and performance were also welcome and considered, intending to provide recent developments within sports sciences research.

A psychophysiological understanding of sports, exercise, and physical activity allows one to construct a bridge between physiological stress and mental wellbeing. In actuality, researchers are still required to provide evidence-based knowledge to coaches and sports scientists to highlight the relationship between training and competition demands, which are related to performance and wellbeing.

In this Research Topic, it is possible to understand that physical exercise can enhance the subjective wellbeing of older adults through sense of meaning in life and self-esteem (Chen et al.). Thus, their perceived competence and sports confidence traits significantly improved after psychological skills training (Lee et al.). Coach leadership in creating a safe environment provides a much better platform to prepare for a pre-crisis stage (Lee et al.). The most positive factor during preparation for a major competition was the high amount of time focused on technical–tactical training (Paludo et al.). Physiological and physical evaluations at different stages and monitoring and control of training and subjective feedback scales are important for injury prevention (Zhao and Jowett). Youths' mathematical skills and performance are related to fine motricity and special visual motor skills (Flores et al.). Understanding psycho-social factors, such as communication, empathy, cohesion, and competition performance, is essential for successful athletic performance (Lee et al.). Another study explored the influence of social support and proactive personality on the anxiety of college students in the post-pandemic era (Zhao and Jowett). A revised version of the Sport Courage scale was created and presented good reliability and validity, and, based on evidence-based information, it can be used as a measurement tool for Chinese athletes. A review study examined published articles concerning sports leadership over the last 30 years, using bibliometric analysis to explore the intellectual base and structural relationships among relevant research components (Flores et al.). A revised version of the Physical Education Grit Scale is presented in this Research Topic and it can now measure Chinese athletes' physical education grit because of its reliability and validity (Paludo et al.).

This Research Topic showed that to comprehend the intricate interplay between the mind and body during physical activity, exercise, and sports, a psychophysiological approach can be seen as facilitator. This approach acknowledges the interdependence of psychological and physiological elements and their potential influence on both sports performance and general health. Understanding the function of stress and physical demands in physical exercise is crucial to comprehend motor behavior. Psychological factors, such as stress, motivation, and anxiety, can have a detrimental impact on sports performance. The importance of individual variations in physical and athletic performance is another crucial component of the psychophysiological approach. Chronic and acute exerciseactivity responses can be influenced by a variety of factors, including personality traits, motivation, and past experiences. Coaches and trainers may better personalize training plans to each athlete's demands by being aware of these unique distinctions. Finally, the psychophysiological approach offers a thorough framework for comprehending the intricate interactions between the psychological and physiological aspects of physical activity, exercise, and sports. Athletes and coaches may enhance performance and advance general health and wellbeing by approaching training and competition holistically.

## Author contributions

PF and DM contributed to conception and design and wrote sections of the manuscript. DP and JT wrote the first draft of the manuscript. All authors contributed to manuscript revision, read, and approved the submitted version.

